# Investigation of the Structure-Forming Potential of Protein Components in the Reformulation of the Composition of Edible Films

**DOI:** 10.3390/ma17040937

**Published:** 2024-02-17

**Authors:** Monika Janowicz, Sabina Galus, Karolina Szulc, Agnieszka Ciurzyńska, Małgorzata Nowacka

**Affiliations:** Department of Food Engineering and Process Management, Institute of Food Sciences, Warsaw University of Life Sciences–SGGW, 159c Nowoursynowska St., 02-776 Warsaw, Poland; monika_janowicz@sggw.edu.pl (M.J.); karolina_szulc1@sggw.edu.pl (K.S.); agnieszka_ciurzynska@sggw.edu.pl (A.C.)

**Keywords:** edible films, biodegradable packaging, gelatin, structure-forming properties

## Abstract

To optimize the functional properties of edible films or coatings, mixtures of several ingredients are used, including food processing by-products. In this way, pectin from fruit pomace, whey proteins from whey as a by-product of rennet cheese production, and gelatin from by-products of the processing of slaughtered animals can be obtained. The aim and scope of the investigation were to verify the hypothesis of the research, which assumes that the addition of beef broth to edible gelatin films will affect the gelation processes of the tested film-forming solutions and will allow for the modification of the edible properties of the films obtained based on these solutions. Measurements were carried out to determine the visual parameters, mechanical strengths, surface and cross-sectional structures, FTIR spectra, thermal degradation rates, and hydrophilicities of the prepared gelatin films. The water content, water vapor permeability, and course of water vapor sorption isotherms of the films were also examined, as well as the course of the gelation process for film-forming solutions. The addition of broth to film-forming solutions was found to increase their opacity and color saturation, especially for the ones that were yellow. The films with the addition of broth were more uneven on the surface and more resistant to stretching, and in the case of the selected types of gelatins, they also formed a more stable gel. The broth increased the hydrophilicity and permeability of the water vapor and reduced the water content of the films. The addition of broth enables the practical use of edible films, but it is necessary to modify some features.

## 1. Introduction

Nowadays, food producers place great emphasis on reducing the negative impact on the natural environment caused by the use of plastic packaging. The activities in this direction focus mainly on modifying the properties of packaging to be able to recycle them, but also focuses on the attempts to replace conventional plastics with biodegradable ones [1]. Many studies are being carried out to create biodegradable packaging in the form of an edible film (a sheet of previously produced material in which food is wrapped) or an edible coating (a form adhering to food, created by immersing the food product in a solution which, after drying, creates a coating) from various types of components; the ingredients most commonly used that are of natural origin are polysaccharides, lipids, and proteins [2]. The group of ingredients whose task is to ensure the appropriate functional properties of edible films and coatings includes polysaccharides, such as cellulose and its derivatives; starches; chitosan; pectin; lipid substances, such as beeswax, most often beeswax or shellac; and a wide range of protein substances, such as whey proteins, wheat gluten, and gelatin [3,4]. The additional use of production by-products is attractive to food producers, because it reduces the waste produced by companies, reduces the problem of waste management (in the case of the meat industry, it is often environmentally hazardous waste), and increases the range of products and semi-finished products produced by companies, which may contribute to increasing their potential importance in the food market [5,6].

From a chemical point of view, hydrocolloids are defined as long-chain biopolymers, mainly composed of polysaccharides but also of proteins (e.g., gelatin), having the ability to form viscous suspensions and gels in water. In food technology, hydrocolloids are used, among other purposes, as thickeners, stabilizers, gelling substances, and foam-maintaining substances. These substances can also serve as substitutes for fat in dairy products or gluten in bread [7]. Some hydrocolloids also serve as emulsifiers in products that form thermodynamically unstable systems, such as emulsions. In addition to the purposes described, these substances can also create thin films that can be used successfully as edible films or coatings [8].

Currently, a lot of research is being carried out to extend the shelf life of food using edible films. The research mainly focuses on enriching the compositions of the matrices of edible structures with essential oils or nanoparticles. The addition of these types of substances has been proven to effectively extend the time for which food is suitable for consumption. Examples of solutions that use the enrichment of edible films with essential oils or nanoparticles include pears covered with a cross-linked cassava starch film enriched with starch nano-crystals [9], shrimps covered with a coating made from sweet potato starch enriched with thyme essential oil [10], pork covered with a film made from a chitosan nanoemulsion enriched with thyme essential oil or thymol [11], and crushed kiwi fruit covered with a gel based on aloe enriched with lemon essential oil [12]. The examples given illustrate that the research on shelf-life extension focuses on a wide range of foods, achieving satisfactory results.

Gelatin is a peptide substance belonging to the group of hydrocolloids, characterized by amino acid chains containing repeating sequences of proline, hydroxyproline, and glycine. Gelatin stands out from other hydrocolloids in its ability to form thermo-reversible gels and in the ability to form these gels at a concentration of just 1%. The temperature determines whether the gelatin solution is in the form of a liquid (sol) or solid (gel), and by changing the temperature, it is possible to reversibly change one state of the solution into another [13]. The described transformation of the gel into a sol occurs at a temperature of approximately 30–40 °C, allowing gelatin gels to dissolve in the mouth during eating. How gelatin will react to temperature changes depends on its chemical structure. Gelatins from different sources will be characterized by different molecular weights, spatial structures (especially in terms of how the α-chains join together to form larger structures), and amino acid compositions. These features will also affect properties such as the gel strength and viscosity [14]. Moreover, it has been proven that the densities of proline and hydroxyproline will affect the thermostability of a given type of gelatin. This is because the zones in the gelatin molecule rich in these amino acids are responsible for creating bonds between individual polypeptide chains, thus stabilizing the structure of the entire molecule [15]. Due to the wide range of possible uses and its properties, gelatin is widely used in food technology as well as other industries. It is used, among other purposes, as a gelling ingredient in the production of cosmetic products such as face creams, body lotions, sunburn prevention milks, and shampoos. In medicine and pharmacology, gelatin is mainly used as a substance that allows for the encapsulation of medicinal substances and dietary supplements [16]. Gelatin is also used as a carrier for intravenous injections; it is also applied in the form of a modern compress aimed at accelerating the healing of swelling and skin wounds. In food technology, gelatin is widely used as a result of its structure-forming properties, so it is used in the production of sweets and confectionery to create gels, especially those stable at lower temperatures. It is also used in the wine and beer industry to clarify wine and beer [17]. Additionally, gelatin can be consumed as a component of a recovery diet for patients who have experienced bone problems such as fractures [18,19].

Many solutions have been developed to find practical applications for edible films and gelatin-based coatings. Researchers who work on such solutions usually focus their efforts on constructing edible packaging designs that would achieve an added value, usually by including substances in the film-forming material that are intended to extend the shelf life of the product [20]. This usually comes down to the design of active packaging containing substances that are intended to significantly reduce the rates of physicochemical and microbiological transformations that occur in food. When designing packaging with edible gelatin films, their mechanical and physicochemical properties should be taken into account [21]. It is easier to produce packaging using fossil raw materials, which are used for this purpose and have satisfactory mechanical properties, than from more ecological materials, which constitutes a great challenge in the potential replacement of conventional polymers with biodegradable ones in packaging. Packaging made from edible film should include, among other characteristics, a satisfactory water vapor barrier and the ability to permeate gases such as carbon monoxide (IV) or molecular nitrogen, as well as a tensile strength that could match packaging made from non-biodegradable polymers [22,23].

A gel made only from a gelatin solution is usually characterized by a high brittleness. This is an undesirable feature for the production of edible films. Therefore, to give the gel produced in this way elasticity, plasticizers are added. The substances that give plastic properties to edible films are most often polyols, such as sorbitol or glycerol, of which the most common plasticizer is glycerol. This plasticizer increases the flexibility of the polymer chain by reducing the intermolecular forces that occur along its structure. As a result, the films made using plasticizers are more susceptible to elongation but can withstand less force when stretched. The addition of plasticizers to edible films also increases their solubility in water and reduces the water vapor barrier. Furthermore, some studies have shown that the use of plasticizers, such as glycerol, results in more effective water retention in gel systems, thus increasing the flexibility of the entire system [11,24,25].

The method of expressing the power (strength) of forming the polymer matrix used to characterize gelatin is a direct result of the Bloom test, which allows for the strength of the resulting bond of polymer structure components to be determined as the Bloom value. The gelatins described by the Bloom test are determined by numerical values that indicate the mass of the piston that caused the collapse of the structure created using gelatin of a given type. This means that the higher the Bloom index, the harder the polymer matrix produced. The Bloom value is also directly proportional to the ability of the selected type of gelatin to retain water in the form of the produced polymer structure. This property is referred to in the literature for any type of hydrocolloid, not only gelatin, as the gelling capacity. One of the main factors determining the Bloom value is the molecular weight of the type of gelatin used, which is the result of the raw material from which it is obtained, as well as the pre-treatment and preparation method [26]. The hardness of the resulting polymer matrix will vary depending on the raw material from which it was obtained and the parameters of the gelatin extraction process from this raw material. In industrial practice, depending on the source of collagen, the pre-treatment, and the method of obtaining gelatin, it is possible to obtain a hydrocolloid with a Bloom value from 50 to 300. Of the values mentioned, gelatin with a Bloom value below 150 is considered to be not very hard (“low Bloom”), being characterized by values in the range of 150–220 is considered medium hard, while a Bloom value in the range of 220–300 is considered very hard. Therefore, the Bloom value of gelatin is inversely proportional to the temperature used in the preparation process. In industrial practice, this translates to the fact that the initial batches of gelatin extracted from the raw material will be characterized by a higher Bloom value [27,28].

The structures obtained based on polymer matrices from gelatins and characterized by different Bloom values will have different physical, functional, and utility properties. In addition to the obvious differences in the hardness of the created polymer structures in the form of films and coatings, they will also be resistant to hydrolysis, which also results in a higher storage stability, and this also applies to a greater resistance to higher temperatures, which is important when products are stored under distribution conditions in places with a warmer climate. Typically, polymer matrices containing gelatin with a higher Bloom index are characterized by a greater weight and mechanical strength [19]. The Bloom value, together with factors such as the concentration of the structure-forming substance or the spatial structure of the matrix, influences properties such as the texture and viscosity of the film and film-forming solution. Gelatin, characterized by a higher Bloom value, will also have a higher melting and gelation temperature, and will also have a shorter binding time of the film or coating components to the structure of the polymer matrix being created [29]. The final product made from gelatin with a higher Bloom value will be more neutral in terms of its odor and taste. The greater gelling power of gelatin also means that it will be necessary to use smaller amounts of it to achieve a polymer structure with the required hardness in the final product, allowing the cost of producing a food product that is already at the design stage to be minimal [28].

The work aimed to analyze the possibility of using the stabilizing, gelling, thickening, binding, and emulsifying properties of long-chain biopolymers, mainly proteins (e.g., gelatin), with the ability to form dense suspensions and gels in water as potential structure-forming additives in the creation of edible films and coatings based on beef broth. Due to the above, a research hypothesis was established, the verification of which is based on the assumption that the addition of beef broth to edible gelatin films will affect the gelation process of the tested film-forming solutions and will allow for the modification of the edible properties of the films obtained based on these solutions. At the same time, it is expected that the gelatins with various degrees of gelation used will allow for the composition of edible foil to be developed in such a way as to enable their practical use in the future as an integral part of the packaging of ready meals in terms of the added value to the finished product, and the stabilization of the product for its positioning in the packaging for appropriate exposure. 

The scope of the research, according to the purpose, assumed the verification of the hypothesis based on measurements of the visual parameters, mechanical strengths, surface structures and cross-sections, FTIR spectra, thermal degradation rates, and hydrophilicities of the foils, as well as the water content, permeability, course of water vapor sorption isotherms of the foil, and the course of the process gelation of film-forming solutions.

## 2. Materials and Methods

### 2.1. Materials

The research material consisted of edible films made from gelatin, beef broth (containing, according to the manufacturer’s specifications, on average, about 5% protein), and glycerol acting as a plasticizer. Pork and beef gelatin, differing in Bloom index, were used to produce the film. All films were obtained based on solutions with a gelatin concentration of 8%. The full list of the components included in the film-forming solutions and edible films obtained on their basis, along with their percentage share, is presented in Table 1.

To produce edible films, 200 cm^3^ of film-forming solution was prepared each time. A total of 16 g of powdered gelatin of each type was measured, depending on the test sample being prepared. The prepared gelatin was then poured into approximately 150 cm^3^ of distilled water or broth and heated using an RCT basic magnetic stirrer [IKA Poland Sp. z o.o., Warsaw] to 60 °C at 600 rpm. The solutions were mixed at a constant temperature for 5 min to ensure that the gelatin was completely dissolved. The solutions were then cooled to a temperature of 50 °C, and glycerol was added to them in the amount of 50% of the added gelatin (8 g) and supplemented to 200 cm^3^ using distilled water or broth, depending on the experimental variant. The solutions prepared in this way were placed again in the magnetic mixer, and to properly standardize the film-forming matrix, they were heated to 40 °C while stirring at 600 rpm. After thoroughly mixing all the ingredients (approximately 5 min), the film-forming solutions were poured onto sheets to obtain thin structures of edible films, using a ZAA 2300 applicator (ZEHNTNER, Sissach, Switzerland). The sheets were then placed in the SUP-65 WG dryer (WAMED Manufacturer of Medical Equipment, SSP, Warsaw, Poland) for 48 h at 30 °C to condition them to obtain a stable and solid edible film. After 48 h, the edible films were removed from the sheets and the necessary markings were made to determine the selected properties of the obtained structures.

### 2.2. Properties of Edible Gelatin–Broth Films

#### 2.2.1. Water Content

The water content was determined based on the dry matter content, which was determined by weight. The determination of each variant of the edible films tested was carried out in three cases of repetition.

The water content (*u*, g H_2_O/g_d.m._) was calculated from the formula:

(1) $$u=\frac{m_{1}-m_{2}}{m_{2}},$$

where *u:* water content (g H_2_O/g_d.m._); *m*_1_: the mass of the sample before drying (g); and *m*_2_: the mass of the sample after drying (g).

#### 2.2.2. Thickness

Film thickness was tested using a ProGage thickness gauge [Thwing-Albert Europe, Deerlijk, Belgium). The measurement was performed with an accuracy of 0.1 μm. Thickness tests for each variant of edible film were performed in ten repetitions.

#### 2.2.3. Opacity

The measurement of the opacities of the tested edible films was performed using a UV–visible Thermo Scientific Evolution 200 series spectrophotometer [Thermo Fisher Scientific Inc., Walthman, MA, USA]. The opacity was calculated based on an absorbance measurement at a wavelength of 600 nm. The measurement was performed in ten repetitions for each type of material tested.

#### 2.2.4. Mechanical Properties

Mechanical property testing was performed using a TA XT2i texture meter (Stable Micro Systems Ltd., Surrey, UK). The coating tensile strength test was performed according to ASTM D882-02. A minimum of 10 repetitions were performed for each type of coating. The tensile strength (*TS*) elongation at break (*E*) was calculated according to the following equations:

(2) $$TS=\frac{F_{max}}{A},$$

where *F_max_*: a force causing a rupture of the shell [N] and *A*: across-sectional area of the shell before the tensile test (mm^2^)

(3) $$E=\frac{∆l}{l_{0}}\cdot 100$$

where ∆*l*: the elongation of the sample at which the film was broken [mm] and *l*_0_: the initial length of the sample before the tensile test (mm).

#### 2.2.5. Water Vapor Permeability

The water vapor permeability through the films was calculated based on the gravimetric method presented by Debeaufort et al. [30], with 50–100% relative humidity differentials using the KBF 240 climatic and water chamber (Binder, GmbH, Tuttlingen, Germany). Linear regression was used to calculate changes in the masses of the samples over time, omitting the first measurements to stabilize the process conditions. The determination was performed in triplicate. The permeability of water vapor was calculated from the formula:

(4) $$P=\frac{∆m\cdot e}{A\cdot ∆t\cdot ∆p}$$

where *P*: water vapor permeability (g/(m·s·Pa)); Δ*m*/Δ*t*: the loss of sample mass over time (g·s^−1^); e: coating thickness (m); *A*: permeation area (0.000804 m^2^); and Δ*p*: thepressure difference under the film and outside (Pa).

#### 2.2.6. Color

The color measurements of the tested edible films were performed using a Chroma Meter CR-400 [Konica Minolta Co., Ltd., Tokyo, Japan] colorimeter in the CIE L*a*b* color system by measuring the trichromatic components L*, a*, and b*, where L* means color brightness, a* is the color axis from green (−a) to red (+a), and b* is the color axis from blue (−b) to yellow (+b). The films were placed on the measuring plate of the colorimeter, and their color was measured in ten repetitions for each type of film. Color measurements were conducted for the control sample, and based on the obtained values of L*, a*, and b*, the average values were calculated, which constituted the formula for measuring the color of the test sample. After the measurement, the films were characterized using the following parameters: total color difference (Δ*E*), color saturation (C*), and color tone (H*).

#### 2.2.7. Thermal Properties

A thermogravimetric analysis and differential thermogravimetric analysis were performed using a TGA/DSC analyzer [Mettler Toledo, Greifensee, Switzerland] in the presence of nitrogen. Edible film pieces weighing approximately 8–9 mg were weighed into a 70 μL crucible made of aluminum oxide. The sample was heated from 30 to 600 °C at a rate of 5 °C/min. with a simultaneous gas flow in the combustion chamber of 50 mL per minute. Based on the tests performed, curves of heat flux changes as a function of temperature and mass loss were obtained. The STARe program was used to interpret the results, allowing us to determine the temperature of the thermal transformation and the mass loss of the sample.

#### 2.2.8. FTIR Spectroscopy

Fourier-transform infrared spectra for the tested edible films were obtained using a Cary 360 FTIR spectrophotometer [Agilent Technologies, Santa Clara, CA, USA], using total reflection (ATR) and single reflection techniques. The systematicity of the test was optimized because of the continuous pressure of the material against the crystal using a pressure clamp. The spectra were taken with a resolution of 4 cm^−1^ in the absorption range from 4000 to 650 cm^−1^. Three repetitions were used for the spectral measurement of one sample.

#### 2.2.9. Testing the Temperature of Polymer Matrix Formation: Gelation Temperature

Measurements were made using a MARS 40 oscillatory rheometer [Haake, Germany] on a plate–plate measurement system with a P60/Ti sensor. Immediately before the measurement, the solutions were conditioned at 40 °C. Then, to perform the measurements, the film-forming solutions were applied using an automatic pipette to the rheometer plate in the amount of 2.8 mL. The plates were then heated under controlled tension and oscillation at a frequency of 1 Hz for 5 min to reach the initial test temperature (35 °C). The next step in the study was to cool the solutions on rheometer plates at a linear rate of 2 K/min to a temperature of 10 °C. The final temperature was maintained for 5 min. The results were recorded using Rheowin Job Manager [Haake Technok GmbH, Vreden, Germany]. The gelation temperatures for the tested solutions were determined from graphs based on the elastic modulus G′ and viscous modulus G″ as the intersection points of the G′ and G″ modulus curves.

#### 2.2.10. Contact Angle Testing

The contact angle analysis was carried out by applying a drop of distilled water to the edible film tested. The measurements were performed using an OCA 25 goniometer [DataPhysics Instruments GmbH, Filderstadt, Germany]. Contact angle tests consisted of sprinkling 10 μL of distilled water onto the top surface and onto the bottom surface of the film. The measurement was performed in 5 repetitions for each type of surface and each type of film. The results were collected and processed using SCA20 software [DataPhysics Instruments GmbH, Filderstadt, Germany].

#### 2.2.11. Structure

The structures of the edible films tested in all obtained cases were documented by electron microscopy photographs using a TM-3000 HITACHI scanning electron microscope [Hitachi High-Technologies Corporation, Tokyo, Japan]. The samples for the structure assessment were prepared in the form of squares measuring 5 × 5 mm. The photos were taken at 500 and 1000× magnification for the cross-section and at 1000× for the surface of the edible films.

### 2.3. Statistical Analysis

A statistical analysis of the results, to determine the impacts of the type of gelatin and the addition of broth on the properties of the edible films tested, was carried out using STATISTICA version 13.3 (Statsoft Polska Sp z o. o., Kraków, Poland) using a multivariate analysis of variance at the significance level of α = 0.05. Furthermore, in some of the determinations, homogeneous groups were determined using the Tukey test (α = 0.05).

## 3. Results and Discussion

### 3.1. The Influence of the Addition of Beef Broth on the Opacity, Color, and Visual Characteristics of Edible Gelatin–Broth Films

The edible gelatin–broth films used as the research material were characterized by varying opacities and colors. The values of the color parameters and the opacities of the film are presented in Table 2. The analysis of the influence of the addition of beef broth on the appearances and selected characteristics of the edible films produced began with a visual assessment. Based on the visual assessment, clear differences were found between the films made as control tests based on water and the films obtained based on beef broth. The broth-based films were characterized by a much darker color, in shades of yellow–brown. At the same time, no clear differences in the color were observed between the films that differed in the type of gelatin used (Bloom) and those that did not differ in the structure-forming matrix base used (broth or water). No significant differences in brightness were observed between the water-based gelatin films. A similar trend was also observed in the gelatin films made from beef broth, regardless of the type of gelatin used.

The analysis of the color parameters and the opacities of edible gelatin films confirmed the observations discussed above based on the visual assessment. All gelatin films made with water were characterized by a similar brightness, and based on the homogeneous groups determined, did not show a statistically significant (*p* < 0.05) similarity to the films made based on broth (Table 2). The gelatin films produced based on water were characterized by a significantly greater brightness compared with the gelatin films obtained based on solutions using beef broth. Within individual samples (control and research), the Bloom value had an impact on the brightness of the tested samples. However, the films made based on water from gelatins with low Bloom values (PG 180 C and BG 160 C) did not belong to the same homogeneous groups as the films made based on broth from gelatins with low Bloom values (PG 180 B and BG 160 B). On this basis, it was concluded that both the type of gelatin used and the presence of broth influenced the brightness of the edible films. This conclusion was supported by the analysis of variance (Table 3), which confirmed the influence of these two factors but did not confirm the existence of an interaction between them. As a result, the highest brightness was observed in edible films without broth and made from gelatin with a low Bloom index (PG 180 and BG 160).

Analyzing the changes in the color coefficient a* of the tested edible gelatin films, it was found that all of the tested samples were characterized by a negative value, which describes the intensity of the green color in the assessed sample [31]. The values of the color coefficient a* that determined the broth-based gelatin films were lower than those for the water-based films, so the broth slightly intensified the green color in the tested samples. Positive values of the color coefficient b* are responsible for the intensity of the yellow color in the materials tested. Edible gelatin films made based on water were characterized by low values of this color chromaticity coefficient, additionally located within one homogeneous group, with no statistically different means. However, the values describing the intensity of the yellow color for edible gelatin films based on beef broth were significantly higher, regardless of the type of gelatin and the structure of the matrix of the tested materials. On this basis, it is true to say that the addition of broth influenced the intensity of the yellow color in the tested samples, which was also confirmed by the analysis of variance (Table 3).

The Δ*E* value was calculated based on the trichromatic coordinate values describing edible gelatin films, both water-based and beef broth-based. The value of the absolute color difference parameter defines how difficult it is to differentiate the tested structures in terms of their color, depending on the structuring plasticizer used (water/beef broth). The parameters of the absolute color difference Δ*E* for the tested variants of edible gelatin films, calculated based on the obtained measurement results, assumed very high values, which prove that the colors of the edible gelatin-broth films could be easily distinguished from the water-based films produced, even by an inexperienced observer. A significant effect of the beef broth on color saturation was found. Values characterizing water-based gelatin films, regardless of the type of gelatin used, ranged from 0.95 to 2.32, and no statistically significant differences were found between these samples.

Edible gelatin films made based on beef broth were characterized by a higher color saturation (C*), with the highest saturation characterized by films made from gelatin with the highest Bloom values (PG 280 B and BG 280 B). The influence of the type of gelatin and the presence of broth in the tested films on the color saturation was confirmed by a statistical analysis (Table 3). The value of the color tone parameter (H*) was the lowest for gelatin films made based on water, compared to films made based on beef broth. This means that the addition of broth caused the films to have a color significantly close to yellow-brown. The effect of the type of gelatin used was less pronounced than that of the presence of beef broth but was significant, which was confirmed with the analysis of variance (Table 3).

The opacity of edible gelatin films is a very important parameter, significantly influencing the acceptance of consumers who pay attention not only to the visual values, but also to the functional values of this type of material [32]. Opacity values (*O*) for the tested edible gelatin films are presented in Table 2. The opacities of the edible gelatin films made based on water were similar, regardless of the type of gelatin used to structure the materials. These samples formed one homogeneous group, so there was no statistically significant difference between them. The opacity was greater in the case of gelatin films made with broth, and within this group of materials, the influence of the gelatin used was also significant. The sample made from beef gelatin with the lowest Bloom value (160) with the addition of broth had the highest value of the opacity parameter. The influence of both the broth and the type of gelatin was confirmed using an analysis of variance (Table 3).

### 3.2. The Influence of the Addition of Beef Broth on the Water Vapor Permeability of Edible Gelatin–Broth Films

When designing food packaging, materials are most often used with properties that allow for the migration of moisture from and into the food to the greatest extent possible, so in the case of many food products, the ideal packaging material will have the highest possible barrier properties, relative to water vapor [33]. Gelatin and composite mixtures containing other film-forming substances are potential ingredients from which edible films can be produced, with optimal properties in terms of their water vapor permeability. Jridi et al. [34] proved that the factors that influence the water vapor permeability of gelatin-based films include the origin of the gelatin and the method of obtaining it.

The analysis of variance performed for the tested samples (Table 4) proved that the type of gelatin used did not affect the water vapor permeability, while the use of beef broth as a plasticizing solvent significantly influenced the water vapor permeability of the structure tested, produced in the form of a film. It was observed that gelatin films based on beef broth were characterized by a higher water vapor permeability compared to films based on water; however, this relationship was confirmed by a statistical method only in the case of structured films using beef gelatin with a Bloom value of 160. The research results obtained can be compared with the results obtained by Benbettaïeb et al. [35], who observed that in a humid environment, gelatin–chitosan films swelled by absorbing water, and as the swelling progressed, the permeability of the tested materials increased. The researchers theorized that this is related to the hydrophilicity of gelatin and the rapidly plasticizing effect caused by water penetrating the film structure, resulting in the loosening of the structure, which facilitates the migration of water vapor. Enriching edible gelatin films with beef broth, which is a mixture of hydrophilic proteins, could have caused an effect similar to that of the previous study.

**Figure 1:**
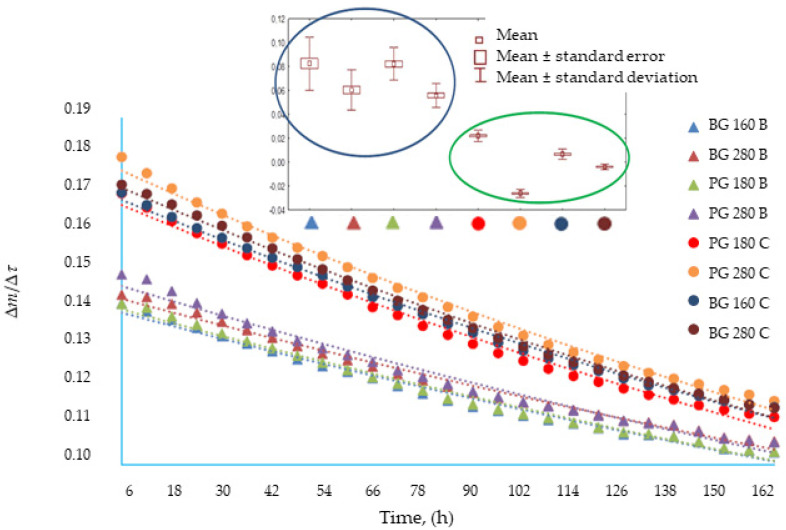
The rate of mass change as a function of time in tests of water vapor permeability through edible gelatin–broth films and the results of Tukey’s reasonable difference of means (RIR) analysis.

The analysis confirmed that both the type of gelatin used and the presence of broth influenced the dynamics of mass changes during the water vapor permeability test through edible gelatin films (*p* < 0.05). Similar average values are shown for films based on broth: PG 180, BG 160, and BG 280 films. Figure 1 also presents a categorized graph for a reasonable difference in means. It was observed that within the group of gelatin films produced based on broth (blue oval in Figure 1), the Bloom value had the greatest impact on the water vapor permeability. Within the water-based films (green oval), no similarities were found between the films made from different types of gelatins. Among the water-based films, a tendency towards lower average values was noticed for films made from gelatin with a high Bloom index. To sum up, the addition of broth to edible films increased the water vapor permeability; the permeability was higher for films with a low Bloom index.

### 3.3. The Influence of the Addition of Beef Broth on the Contact Angle of Edible Gelatin–Broth Films

**Figure 2:**
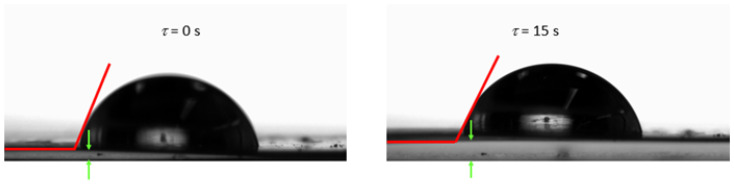
Example of the behavior of water-based gelatin film (PG 180) after applying a drop of water during contact angle testing.

**Figure 3:**
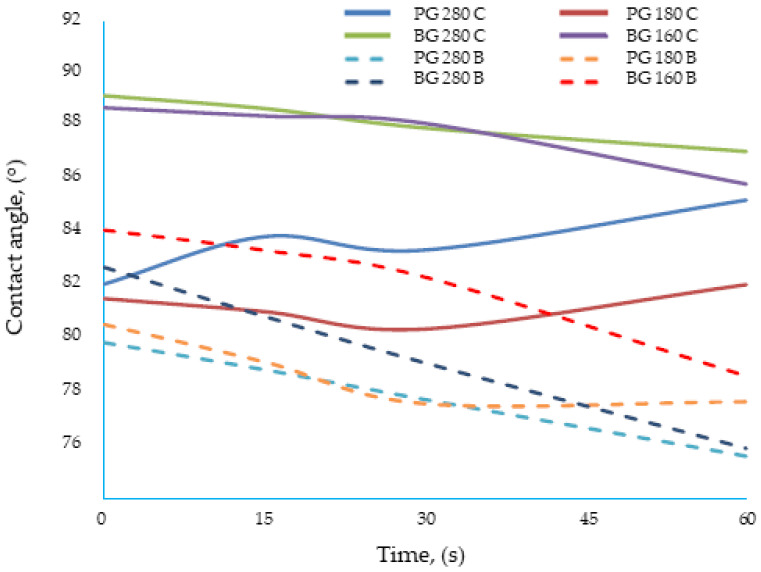
Dynamics of change in the contact angle of the tested edible gelatin–broth films.

For most of the materials tested, the contact angle decreased with time, which was related to the reorientation of polar groups on the film surface [36]. For some edible gelatin films tested, the opposite tendency was observed: the contact angle increased with time. However, this was not the result of drops penetrating the structure of the film matrix, but of “swelling” of the material surface (Figure 2). The wetted surface of the film rose upward due to contact with water, which made the accurate measurement of the contact angle difficult. In this study, the gelatin and new bouillon-gelatin films were characterized by different wetting angles, ranging from approximately 78–89°, depending on the side of the film onto which the drop was applied (Figure 3). These are the values that allow us to conclude that all of the gelatin films tested had a relatively poor hydrophobicity. This statement was made based on a comparison of the edible gelatin films discussed, based on the results obtained in this study, with films based on pure starch [37], which had a contact angle of approx. 70°, which then rapidly decreased to approx. 40° during 60 s of contact of the film surface with a drop of applied water.

The broth films in this study were less hydrophobic than the films studied by Cheng et al. [36]. These were gelatin–starch films containing a strongly hydrophobic component, i.e., wax. In the study mentioned above, the contact angle of edible films immediately after leaving the drop was 102° and decreased slightly over time. Until recently, it was assumed that if the water-wetting angle of the surface of the edible films was greater than 65°, the film was hydrophobic, while if the angle was less than 65°, the film was hydrophilic. However, it is currently assumed that a film is hydrophobic if the water contact angle is above 90°, and it is hydrophilic if the contact angle is below 90° [31]. Using the most current criterion, the edible gelatin films tested in this experiment should be classified as hydrophilic films.

The addition of broth, the type of gelatin used, and the combination of both factors influenced the value of the water contact angle of the edible gelatin films tested, which was confirmed by the analysis of variance. Films made from different types of gelatins were characterized by an ambiguous effect, from the addition of beef broth, on the change in the contact angle. The films made from beef gelatin were very similar to each other. Four groups were distinguished (letter designations (^a–d^) were assigned to the coating designations in one homogeneous group) from the samples with a similar hydrophilic character: (^a^) BG 160 B, BG 280 B; (^b^) BG 160 C, BG 280 C; (^c^) PG 180 B, PG 280 B; and (^d^) PG 180 C, PG 280 C. This similarity was best illustrated by the BG 280 B and BG 160 B films (Figure 3), because the dynamics of change and the values of the contact angles of both films were very similar. The BG 280 and BG 160 films, when water drops were applied, showed higher contact angle values, close to 90°, if they were made based on water. The value of contact angles is much lower for the gelatin films BG 280 and BG 160 (approx. 82°), which were made based on broth. To sum up, for films made from beef gelatin with Bloom values of 280 and 160, the addition of broth increased the hydrophilicity of the tested structures.

### 3.4. The Influence of the Addition of Beef Broth on the Course of Thermal Changes during Thermogravimetric Analysis (TGA) of Edible Gelatin–Broth Films

**Figure 4:**
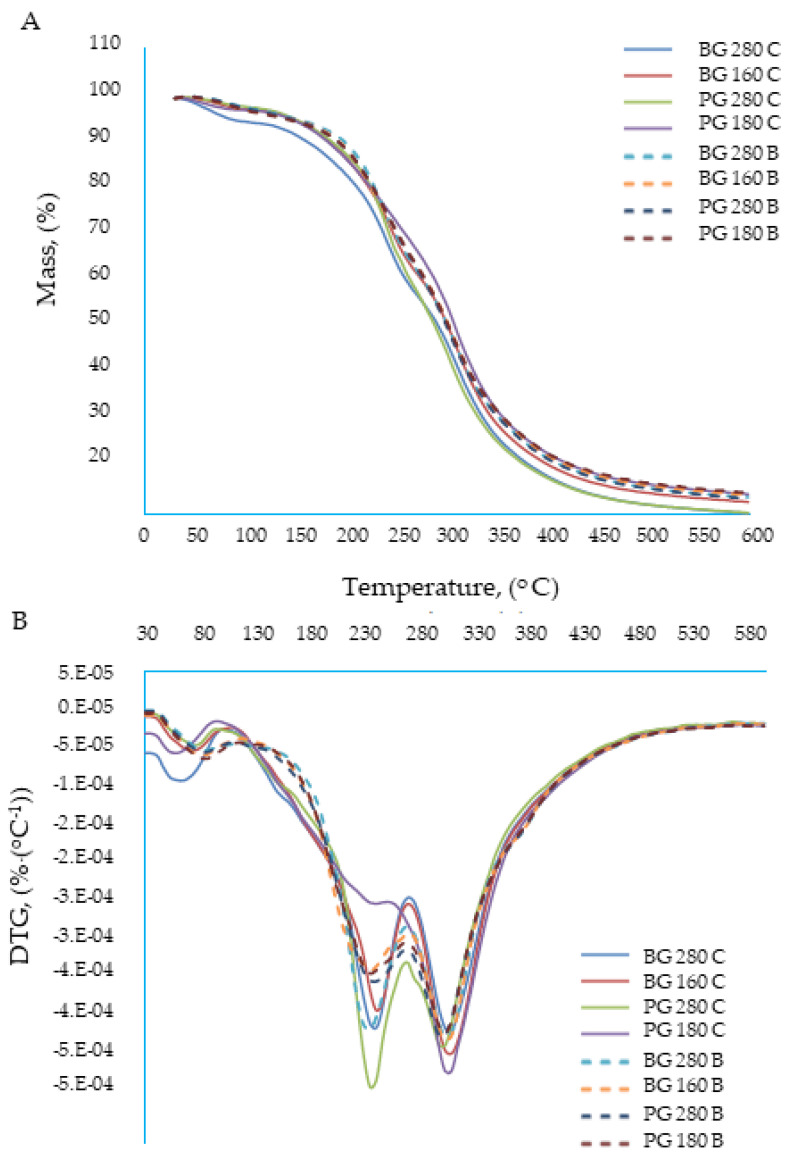
Thermogravimetric analysis of edible gelatin–broth films. (**A**) TGA curves; (**B**) DTG curves.

Thermal analysis methods provide a range of information regarding changes in the selected properties of substances under the influence of temperature that change in a specific manner. Among various materials, organic compounds, both low- and high-molecular weight, constitute one of the groups of materials most commonly studied using thermal analysis methods. The basic importance for the characterization of organic compounds is the determination of their thermal parameters, such as the glass transition temperature, melting point, decomposition temperature, specific mass losses, or polymorphic transformations; they often determine the quality of the product. Research on the determination of the heat of fusion, crystallization, polymorphic transformations, and specific heat is of fundamental importance. These parameters refer to both the organic materials themselves and their structures and origins, i.e., the methods of production and processing. Currently, there is a great interest in nanomaterials, including polymer composites and nanocomposites with shaped thermal stabilities. The assessment of their properties using thermal analysis methods allows for determining the roles of the individual main components and additives, as well as some structure (nanostructure) vs. structure relationships. T properties of composite polymers structured with gelatin based on beef broth were analyzed. An analysis of the ongoing transformations enables the detection of volatile decomposition products, the analysis of residues, and the determination of basic kinetic parameters. For technological and operational reasons, the examination of the degradation process is of fundamental importance from two perspectives: The first concerns the problem of stabilizing the polymer to obtain new materials with desired thermal properties that are capable of meeting the requirements of modern materials engineering. The role and operation of the stabilizer may be more effective as the mechanism of the degradation process is taken into account in its design. The second concept of research on the degradation process focuses on the problem of the decomposition or reuse of bioplastic waste. Both issues are not mutually exclusive, but rather complementary; therefore, a thermal analysis was carried out using beef broth-based composite gelatin films to optimize their features as packaging materials. A thermogravimetric analysis is an assay that provides useful information for substances and mixtures that have the potential to serve as biodegradable packaging materials. The raw materials used to produce the packaging must be thermally stable, allowing the integrity of the product to be maintained during its production and consumption [38]. The TGA and DTG curves are presented in Figure 4A,B, respectively. The curves show that the thermal decomposition of the tested films took place in three stages: The first phase of decomposition occurred at a temperature of 0–120 °C; it was similar in both types of edible films: water based and broth based. However, a slight shift in this decomposition was observed at the temperature of the broth films. The disintegration temperature of the bouillon films was 80.3–83.8 °C, while for the water-based films, it was 60.9–71.9 °C. The disintegration of the first phase was most likely related to the evaporation of the solvent, i.e., water, from the edible films. The greater thermal resistance of gelatin broth films could be related to the lower water content or is strictly related to the more difficult availability (lower activity) of water in these films. The second phase of decomposition occurred in the temperature range of 120–280 °C, and the temperature at which the peak thermal decomposition occurred was 233.4–242.7 °C. No significant differences were observed between the disintegration temperature of the gelatin broth films and the water films. In this phase, the proteins, bound water, and any volatile substances contained in the films most likely disintegrated. Glycerol also decomposed at this temperature [39]. The third phase of decomposition occurred in the temperature range of 280–400 °C, with the peak of the thermal decomposition set at a temperature that oscillates (for all types of edible films) around 305 °C (302.3–310 °C). This value corresponded to the breakdown of gelatin [40].

### 3.5. The Influence of the Addition of Beef Broth on the Course during Infrared Spectroscopic Analysis (FTIR) of Edible Gelatin–Broth Films

**Figure 5:**
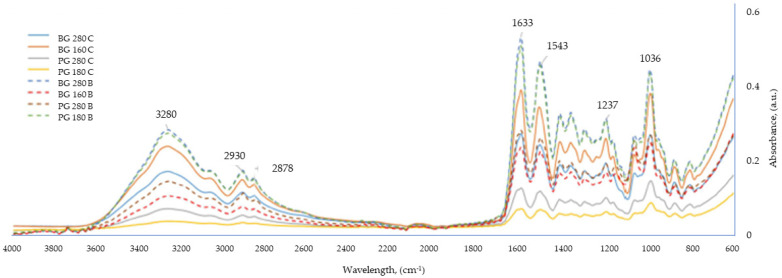
FTIR analysis of edible gelatin–broth films.

FTIR spectroscopy is a method that allows for the qualitative and quantitative definition of chemical bonds occurring in multicomponent systems, such as edible films. The method is often used in the physicochemical analyses of edible films based on polysaccharides and proteins [41]. Figure 5 shows the FTIR spectra for the edible films tested. All analyzed samples presented a very similar spectrum in terms of the peaks occurring at similar wavelengths; the value that differentiated the tested films was the intensity of the absorbance of waves at given wavelengths. The greatest similarities, in terms of absorbance intensity, were found in films made from different types of gelatins but coming from the same type of raw material (beef or pork) and belonging to the same group in terms of the presence of broth. On this basis, four groups of samples were distinguished (letter designations (^a–d^) were assigned to the coating designations in one homogeneous group), showing very similar FTIR spectra: (^a^) BG 160 B, BG 280 B; (^b^) BG 160 C, BG 280 C; (^c^) PG 180 B, PG 280B; and (^d^) PG 180 C, PG 280 C.

It was observed that the Bloom value did not have a strong influence on the FTIR spectra of the edible films tested. The origin of the gelatin used to produce the film (beef/pork) and the presence of broth in the film had a stronger influence.

In Figure 5, six particularly important peaks are marked, characteristic of all samples tested. The peak observed at a wavelength of 3280 cm^−1^ most likely corresponded to stretching vibrations, characteristic of the hydroxyl (-OH) and amino (-NH) groups, which are created in the polymer chain of gelatin [42]. Absorption bands in the range of 2850–3000 cm^−1^ are characteristic of -CH stretching bonds. The absorbance peak at a wavelength of 2930 cm^−1^ corresponded to asymmetric stretching vibrations in methyl groups connected to two (-CH^2−^) or three (>CH^−^) carbon molecules, while the absorbance peak at a wavelength of approximately 2878 cm^−1^ came from symmetric stretching vibrations of the methyl group [38]. A high absorbance of the tested samples was recorded at a wavelength of 1633 cm^−1^, which corresponds to stretching vibrations characteristic of carbonyl groups (C = O) contained in the amide residues in the polymer chain of gelatin [36]. The high peak at a wavelength of 1543 cm^−1^ was responsible for deforming N–H group bonds and stretching C–N bonds; high absorbance values at this wavelength are characteristic of compounds containing gelatin. The peaks at a wavelength around 1400 cm^−1^ corresponded to symmetric deformation vibrations of CH_3_ [43]. Higher peaks at this wavelength were responsible for the higher hydrophobicity of the sample. The highest peaks at a wavelength of approx. 1400 cm^−1^ were recorded in films based on the broths of both beef gelatins, so these samples were characterized by the most hydrophobic character. However, the least hydrophobic, based on the size of the absorbance peak, turned out to be the water-based films made from both pork gelatins. The peak at the wavelength of 1237 cm^−1^ corresponded to the vibrations of the N–H and C–N bonds belonging to the bound amide residues [42]. The absorption band with a peak at a wavelength of approximately 1036 cm^−1^ is characteristic of the C–O stretching vibrations present in glycerol molecules [36,44].

### 3.6. The Influence of the Addition of Beef Broth on the Structure of Edible Gelatin–Broth Films

The type of raw material used to produce edible gelatin films has a significant impact on the appearances of the cross-sections and matrix surfaces of the tested structures. Inequalities and non-uniform surface structures result in unfavorable mechanical properties of an edible film [43]. 

The use of beef broth for the production of edible gelatin films caused changes in the structures of the surfaces and cross-sections of the tested materials. Table 5 contains photos of the surfaces and cross-sections of all the edible gelatin films tested, assessed in terms of their functional and physical properties in this study. Based on our observations, it was found that the addition of beef broth influenced the appearance of the surfaces of the edible gelatin films tested. When comparing both variants of films (with and without beef broth) made from the same type of gelatin, it was noticed that the surface of the matrix structure made based on water was much more uniform compared to the films made based on beef broth. The effect of the addition of broth on the appearance of the cross-section was less pronounced. In some cases (e.g., PG 180 C and PG 180 B films), it was possible to notice a difference in how the films laid on the cross-section. In the case of gelatin films made based on water, their layers were arranged parallel to each other, one on top of the other, while the film made based on broth created a more uniform spatial form, and no layer formation was observed. This observation was not the case for films made from other types of gelatins. This could be due to the different properties of the protein structure-forming components used, but it could also be due to the different fragments from which the samples were taken for testing. Films made from the water-based gelatins PG 180 and BG 160 showed the greatest irregularities in their cross-sections, so they should also be characterized by the lowest tensile strength. In their study, Peng et al. [43] proved that films that were characterized by a greater irregularity in the structure of the cross-section were characterized by a higher water content. A similar relationship was noticed in this study. Films made based on the water of the gelatins BG 160 and PG 180 were characterized by the highest corrugation in the cross-section, and in the water content test (Table 5), they had the highest water content.

### 3.7. The Influence of the Addition of Beef Broth on the Mechanical (Tensile Strength (TS) and Relative Elongation (E)) and Thermal (Film-Forming Matrix Formation Temperature–Gelation Temperature (Tg)) Properties of Edible Gelatin–Broth Films

#### 3.7.1. The Influence of the Addition of Beef Broth on the Mechanical Properties (Tensile Strength (TS) and Relative Elongation (E)) of Edible Gelatin–Broth Films

The mechanical properties of edible films depend on the density of intermolecular and intramolecular chemical bonds that create the three-dimensional structure of the system. Stronger non-covalent bonds can contribute to the formation of a dense film structure, improving (strengthening) the mechanical strength [45]. The determination of the tensile strength (*TS*) and relative elongation (*E*) are used to determine the strength and ductility of the sample, respectively. These mechanical properties are important in terms of the need for the film to maintain the integrity necessary to produce biodegradable food packaging [46]. The results of the tensile strengths and relative elongations obtained for edible films in this experiment are presented in Table 6.

Films containing broth introduced additional protein components into the edible film, in addition to the gelatin also contained in the control sample, which resulted in the concentrations of protein-type substances in broth films being higher than in water-based films. The analysis of variance showed that both the type of gelatin used and the presence of beef broth had an impact on the mechanical strengths of the edible gelatin films tested. The results presented in Table 6 also suggest that the introduction of an additional protein ingredient, i.e., beef broth, to edible gelatin films (although the protein content is only about 5%), increased the tensile strength of a given type of film. However, this observation was statistically confirmed only in the case of films obtained from two types of gelatins (beef 280 and pork 180). The broth and water equivalents of these films belonged to different homogeneous groups, so there was a statistically significant difference between them. Similar results were observed by Benbettaïeb et al. [35], who proved that increasing the share of gelatin in gelatin–chitosan films, i.e., increasing the share of the protein fraction in the mixture, increases the tensile strength of the edible film. Similar conclusions were put forward by the authors of other works [47,48]. Edible films produced from the gelatins beef 160 and pork 280 did not show a statistically significant difference between the samples produced from the same type of gelatin, differing only in the presence of broth. To sum up, the positive effect of the broth on the mechanical strength of the edible film was observed only in the case of films made from the gelatins beef 280 and pork 180. The effect of the presence of beef broth on the relative elongations of edible gelatin films was more difficult to determine. The films made from the same type of gelatin, differing only in the use of broth as a plasticizing solvent, in each case, belonged to the same homogeneous group; therefore, the use of broth did not have any statistically significant effect on the relative elongations of the edible gelatin films tested. This conclusion is confirmed by the analysis of variance, which also shows that broth does not affect the relative elongations of the edible gelatin films tested. The obtained results were compared with the study conducted by Peng et al. [43], who showed that increasing the concentration of the protein hydrocolloid–gelatin increased the relative elongations in composite edible films, but only at a concentration of 15%. By further increasing the gelatin concentration, the researchers observed a decrease in the relative elongation of the film. There was a difference between the films which differed in the type of gelatin used. Films made from gelatins with a lower Bloom value were characterized by a higher relative elongation, but this inversely proportional relationship was statistically confirmed only in the case of bovine gelatin films made based on water. To sum up, the type of gelatin used had an impact on the relative elongation values of the edible gelatin films tested, but the decisive factor determining the parameter value was not the Bloom value. It is assumed that the decisive factor that had the greatest impact on the value of the relative elongation was the origin of the gelatin (beef or pork) used to produce the edible films.

#### 3.7.2. The Influence of the Addition of Beef Broth on the Thermal Properties (Film Formation Temperature–Gelation Temperature (Tg)) of Gelatin Solutions That Structure the Matrix of Edible Gelatin–Broth Films

**Figure 6:**
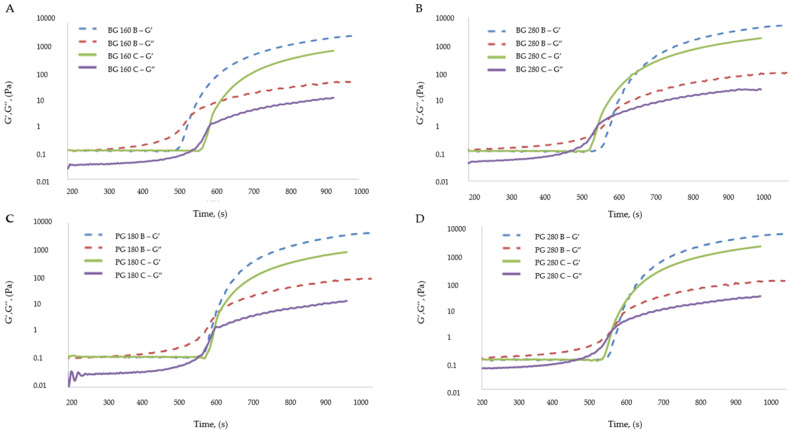
Changes in the elastic modulus (G′) and viscous modulus (G″) for film-forming solutions containing gelatin as a structure-forming component. (**A**) Bloom 160 beef gelatin; (**B**) Bloom 280 beef gelatin; (**C**) Bloom 180 pork gelatin; (**D**) Bloom 280 pork gelatin.

The gelatin solution creates a Newtonian liquid in which, upon cooling, structural changes in the system are initiated. The gelatin solution cooled to a sufficiently low temperature results in a phase transformation. After being further cooled, a permanent gel will be created. This phase transformation is called a sol–gel transformation; the gel formed in this way is a viscoelastic system and has the characteristics of a solid. The characteristics of the gel formed as a result of the sol–gel transformation are influenced by, among others, the temperature after the supercooling of the solution, the type of gelatin used, and the additives added to the solution. The low water content in the solution and additional ingredients in the solution (e.g., added to create fruit jellies) slow down the sol–gel transformation process.Figure 6 presents the gelation temperatures (sol–gel transformations) of the edible gelatin films tested. 

The addition of beef broth to edible gelatin films influenced the gelation temperatures of the film-forming solutions, which was confirmed by the analysis of variance, but a clear description of the effect of beef broth was difficult to define. For solutions containing the gelatins beef 280 and pork 280, the addition of broth caused a decrease in the gelation temperature, so the sol–gel transformation occurred slower in these solutions. For the solution containing the 180 pork gelatin, the addition of broth had the opposite effect: gelation occurred at a higher temperature, so the sol–gel transformation occurred faster. The solutions containing beef gelatin 160, regardless of the inclusion or lack of broth, did not differ statistically in gelation temperature. The type of gelatin used had a much greater impact on the gelation temperatures of the solutions, for which the following tendency was noticeable: solutions that contained and did not contain broth, made from gelatin with a higher Bloom degree (beef 280 and pork 280), were characterized by a higher gelation temperature than solutions that contained gelatin with a lower Bloom degree. This relationship was statistically confirmed in almost every case, both in solutions containing and without broth.

Figure 6 presents the elastic modulus (G′) and the viscous modulus (G″) for solutions containing the following: beef gelatin 160, beef 280, pork 180, and pork 280, in variants presenting water-based solutions (C) or based on beef broth (B). The gelation process involved cooling a given solution from a temperature of 40 °C to a temperature of 10 °C. The gelation processes for water-based solutions were similar for all types of gelatins tested. For solutions based on beef broth, the gelation process was also similar, regardless of the type of gelatin used. The gelation processes of all broth-based solutions differed significantly from the gelation processes of all water-based solutions. Solutions without broth were characterized by values of the viscous modulus that were lower than those of the elastic modulus, even before the gelation process was started. It was noticed that for these solutions, the elastic modulus reached higher values both before and after the gelation process. Graphs showing the course of the gelation process of water-based solutions present two intersection points of the viscous and elastic moduli, the first intersection point being interpreted as the actual gelation temperature.

The two points of intersection of the moduli resulted in the formation of a “loop” between them. It is assumed that this “loop” is responsible for the prolonged gelation process, during which the proper gel structure could have been formed due to the cross-linking of the three-dimensional structure of the system. A pure gelatin solution that is cooled from 40 °C to the gelation point is characterized by higher values of the viscosity modulus, which describe the rheological properties of the solution [49]. The film-forming solutions in this experiment contained, in addition to gelatin, glycerol, which was dissolved in the same solution. It is assumed that the presence of glycerol modified the rheological properties of film-forming solutions at temperatures above the gelation point. For all types of gelatins tested, the elastic modulus (G′), after stabilization of the gel form was achieved, reached higher values in the case of solutions based on broth. The elastic modulus in the case of solutions based on beef gelatin 160 was higher for the solution based on broth by 1888 Pa; in the case of solutions made from other types of gelatins, the elastic modulus was higher by a similar value, equal to 2615 Pa on average. A higher G′ value means a higher thermal stability of the gel [50], which translates into the fact that the gel prepared with the addition of broth was characterized by a higher stability than the gel prepared based on water.

## 4. Conclusions

The research carried out analyzed the results of the evaluation of the properties of gelatin food films in terms of the use of beef broth as a solvent to plasticize the matrices of the produced structures, which could be used instead of water to modify the functional properties of the biodegradable materials obtained in the form of film sheets. Edible gelatin films based on beef broth, which is the plasticizing solvent in the matrices of the tested materials, were characterized by a significantly greater opacity compared to materials obtained based on water as the plasticizing solvent. At the same time, a color assessment based on the analysis of chromatic coordinates allowed us to conclude that the presence of beef broth determines the yellow-brown shade of the obtained films, regardless of the type of structure-forming substance and its Bloom gelation characteristics. The presence of beef broth in edible gelatin films resulted in a slight reduction in the adsorption of water vapor from the surrounding environment under the condition of a low ambient relative humidity. The addition of beef broth made the films more hydrophilic and increased the water vapor permeability of the edible films. The films containing broth had a lower water content. There was no significant effect of the addition of beef broth on the thermal stability of the film. Only a higher thermal decomposition temperature of the broth films was observed in the first phase of the thermogravimetric test, which was most likely related to the lower water content. The introduction of beef broth into the matrix that forms the structure of edible gelatin films made the surface of the films more non-uniform. The addition of beef broth to films made from pork gelatin 180 and beef gelatin 160 resulted in less folding of the film in the cross-section. The bouillon films were characterized by the formation of a more stable gel and a higher tensile strength (in the case of beef gelatin 280 and pork gelatin 180), without affecting the elasticity of the film.

The enrichment of edible gelatin films with beef broth changed the physical properties of these films in a way that enabled their practical use in food products. To use edible bouillon films as a stabilizing element that can be used to position a ready dish, it is recommended to use bouillon film made from beef gelatin with a Bloom index of 280. Gelatin bouillon film containing gelatin as a structure-forming substance at the level of 8% was characterized by a higher tensile strength compared to other films tested and created a gel with high stability. After the analysis of the obtained test results, it was found that further research should be carried out on the possibility of modifying the properties of the films using beef broth, especially in terms of determining the melting point of the gel, which will ultimately allow for specifying the parameters that determine the stability of the bouillon films based on gelatin. When designing food products containing gelatin and beef broth, their visual esthetics, low vapor barrier, and high hydrophilicity should be taken into account. Visible features, which are the opacity and a dark, yellow-brown version of the films, may reveal the attractiveness of the product which they coat, therefore belonging to the product design in a key way that the features are not related (e.g., changing the basic color of the finished product due to a yellow-brown exposure or exposure of the product due to the a high opacity of the film). To compensate for the relevant properties of bouillon films, including their strong hydrophilicity and high secondary vapor permeability, their use may occur in the event of the occurrence of production residues with typically hydrophobic consequences, such as waxes, fats, etc.

## Figures and Tables

**Table 1:** Ingredients used to produce edible gelatin–broth films along with the compositions and markings used to discuss the properties of the edible films (codes) produced.

Component/Type of Gelatin Used (According to Bloom Index) and Concentration (%) ^1^	Gelatin (%)	Beef Broth (%)	Glycerol (%)	Code
**Pork gelatin 280** (granulated) with Bloom index 280 (Gelita AG, Germany)	8	0 88	4	**PG 280 C** **PG 280 B**
**Pork gelatin 180** (granulated) with Bloom index 180 (Gelita AG, Germany)		0 88		**PG 180 C** **PG 180 B**
**Beef gelatin 280** (granulated) with Bloom index 280 (Gelita AG, Germany)		0 88		**BG 280 C** **BG 280 B**
**Beef gelatin 160** (granulated) with Bloom index 160 (Gelita AG, Germany)		0 88		**BG 160 C** **BG 160 B**
**Distilled water**	The control sample consisted of edible films prepared with 100% distilled water (marked with code C)			
**Beef broth**	The experimental sample consisted of edible films prepared with 88% beef broth (marked with code B)			
**Glycerol** (purity 99.5%) Avantor Performance Materials Poland S.A. (Gliwice, Poland)	Glycerol was added in the amount of 50% of the added gelatin			

^1^ The ingredients to prepare the film formation solutions were prepared in 200 cm^3^ portions.

**Table 2:** Average values of chromatic color coordinates for the tested edible gelatin–broth films.

Code	L*	a*	b*	Δ*E*	C*	H*	*O*
**PG 280 C**	91.35 ± 0.51 ^c,^*	−0.63 ± 0.06 ^c^	1.73 ± 0.28 ^a^	-	1.74± 0.50 ^a^	−69.89 ± 1.58 ^cd^	0.66 ± 0.05 ^a^
**PG 280 B**	85.41 ± 1.22 ^a^	−1.30 ± 0.04 ^b^	13.48 ± 2.23 ^c^	90.05 ± 31.41	94.24± 29.04 ^c^	−84.30 ± 1.08 ^a^	1.73 ± 0.16 ^bc^
**PG 180 C**	92.50 ± 0.35 ^de^	−0.56 ± 0.04 ^e^	1.25 ± 0.17 ^a^	-	0.95 ± 0.23 ^a^	−65.85 ± 1.45 ^e^	0.82 ± 0.15 ^a^
**PG 180 B**	86.81 ± 0.76 ^b^	−1.36 ± 0.06 ^ab^	11.35 ± 1.63 ^b^	68.98 ± 19.22	66.65 ± 18.19 ^b^	−83.08 ± 0.75 ^ab^	1.55 ± 0.18 ^b^
**BG 280 C**	91.57 ± 0.37 ^cd^	−0.66 ± 0.04 ^c^	1.94 ± 0.19 ^a^	-	2.12 ± 0.4 ^a^	−71.10 ± 0.95 ^c^	0.77 ± 0.08 ^a^
**BG 280 B**	85.38 ± 0.75 ^a^	−1.42 ± 0.03 ^a^	14.38 ± 1.42 ^c^	98.42 ± 21.86	105.36 ± 19.67 ^c^	−84.32 ± 0.63 ^a^	1.85 ± 0.06 ^d^
**BG 160 C**	92.75 ± 0.35 ^e^	−0.77 ± 0.01 ^d^	2.01 ± 0.05 ^a^	-	2.32 ± 0.09 ^a^	−69.12 ± 0.54 ^d^	0.71 ± 0.10 ^a^
**BG 160 B**	87.17± 0.37 ^cd^	−1.38 ± 0.04 ^a^	10.33 ± 0.34 ^b^	53.02 ± 4.32	57.54 ± 3.63 ^b^	−82.60 ± 0.41 ^b^	2.15 ± 0.21 ^c^

The letters a–e in the upper index indicates belonging to homogeneous groups, between which no statistically significant differences were found (*p* < 0.05).

**Table 3:** Analysis of variance for trichromatic color components in terms of the type of gelatin used (Bloom)/the presence of beef broth in the edible film (C = 0%/B = 88%).

				L*				a*				b*			
	**df**	**SS**	**MS**	**F**	***p***	**SS**	**MS**		**F**	***p***	**SS**		**MS**	**F**	***p***
**Bloom**	3	38.7	12.9	27	0.000	0.186	0.062		31.82	0.000	51.103		17.034	12.345	0.000
**C/B**	1	666.1	666.1	1383	0.000	9.816	9.816		5028.43	0.000	2238.835		2238.835	1622.5	0.000
Bloom *(C/B)	3	1.1	0.4	1	0.525	0.101	0.034		17.2	0.000	43.866		14.622	10.597	0.000
				C*				H*				***O***			
	**df**	**SS**	**MS**	**F**	***p***	**SS**	**MS**		**F**	***p***	**SS**		**MS**	**F**	***p***
**Bloom**	3	7582.4	2527.5	11.473	0.000	116.9	39.0		32.3	0.000	0.703		0.234	13.556	0.000
**C/B**	1	12149.2	12149.2	553.557	0.000	4140.1	4140.1		3428.6	0.000	14.102		14.102	815.659	0.000
Bloom *(C/B)	3	7470.2	2490.1	11.303	0.000	47.1	15.7		13.0	0.000	0.931		0.310	17.957	0.000

**Table 4:** Average values of water vapor permeability for the tested edible gelatin–broth films and results of the analysis of variance of water vapor permeability for the type of gelatin used (Bloom)/presence of beef broth in the edible film (C = 0%/B = 88%).

Code	*WVP* (10^−10^ g⋅ m^−1^⋅ s^−1^⋅ Pa^−1^)	Water Vapor Diffusion Equations and Data Fit Factors Experimental (R^2^)		*WVP*–Results of the Analysis of Variance				
				df	SS	MS	F	*p*
**PG 280 C**	5.56 ± 1.36 ^ac^	Δ*m*/Δ*τ* = 0.1794⋅*e*^−0.003^*^τ^* (0.995)	Bloom	1	0.000000	0.000000	2.6869	0.1031
**PG 280 B**	11.80 ± 2.06 ^bc^	Δ*m*/Δ*τ* = 0.1484⋅*e*^−0.002^*^τ^* (0.984)	C/B	3	0.000000	0.000000	29.7574	0.0003
**PG 180 C**	9.97 ± 0.36 ^abc^	Δ*m*/Δ*τ* = 0.1702⋅*e*^−0.003^*^τ^* (0.993)	Bloom *(C/B)	1	0.000000	0.000000	1.9061	0.1926
**PG 180 B**	13.2 ± 1.71 ^b^	Δ*m*/Δ*τ* = 0.1421⋅*e*^−0.002^*^τ^* (0.989)						
**BG 280 C**	7.7 ± 1.24 ^abc^	Δ*m*/Δ*τ* = 0.1748⋅*e*^−0.003^*^τ^* (0.984)						
**BG 280 B**	10.5 ± 1.63 ^abc^	Δ*m*/Δ*τ* = 0.1449⋅*e*^−0.002^*^τ^* (0.986)						
**BG 160 C**	3.25 ± 1.01 ^a^	Δ*m*/Δ*τ* = 0.1715⋅*e*^−0.003^*^τ^* (0.996)						
**BG 160 B**	12.4 ± 2.05 ^bc^	Δ*m*/Δ*τ* = 0.1413⋅*e*^−0.002^*^τ^* (0.987)						

The letters a–c in the upper index indicates that it belongs to homogeneous groups, among which no statistically significant differences were found (*p* < 0.05).

**Table 5:** Surface and cross-sectional structures of the tested edible gelatin–broth films. The documentation of the surface was made at ×100 magnification, and the cross-section was made at ×500.

Code	Surface Structure	Cross-Sectional Structure	Water Content, (gH_2_O⋅g _d.m._^−1^)
PG 280 C	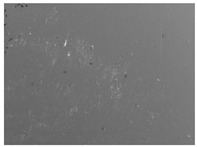	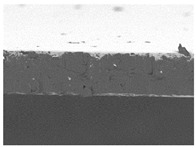	0.1804 ± 0.029 ^b,^*
PG 280 B	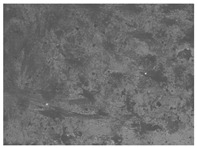	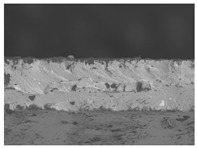	0.1104 ± 0.002 ^de^
PG 180 C	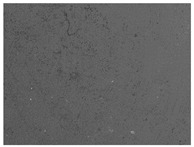	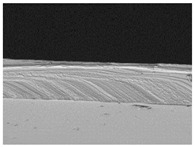	0.2388 ± 0.079 ^a^
PG 180 B	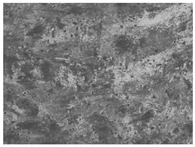	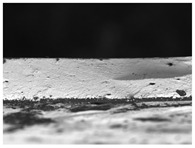	0.1078 ± 0.009 ^de^
BG 280 C	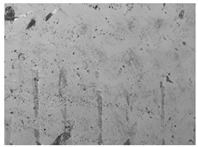	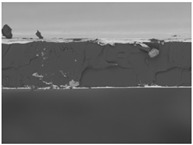	0.2291 ± 0.009 ^c^
BG 280 B	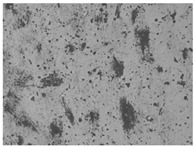	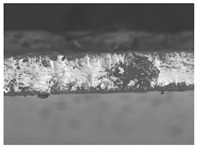	0.1023 ± 0.001 ^e^
BG 160 C	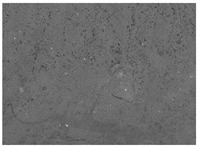	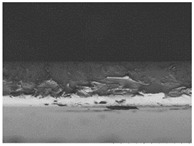	0.2342 ± 0.090 ^a^
BG 160 B	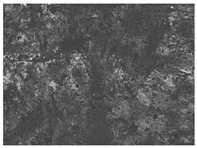	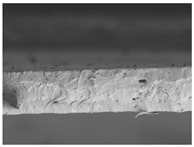	0.1542 ± 0.026 ^cd^

The letters a–e in the upper index indicates that they belong to homogeneous groups, between which no statistically significant differences were found (*p* < 0.05).

**Table 6:** Mechanical properties (tensile strength (TS) and relative elongation (E)) and thermal properties (film-forming matrix formation temperature–gelation temperature (Tg)) of the tested edible gelatin–broth films.

Code	*TS* (MPa)	*E* (%)	T_g_ (°C)
**PG 280 C**	25.14 ± 5.7 ^bc^	20.70 ± 5.0 ^a^	26.76 ± 0.00 ^e^
**PG 280 B**	27.26 ± 8.8 ^c^	23.30 ± 10.1 ^a^	24.94 ± 0.06 ^c^
**PG 180 C**	18.11 ± 2.8 ^a^	33.80 ± 10.7 ^abc^	23.16 ± 0.11 ^a^
**PG 180 B**	25.68 ± 6.0 ^bc^	26.70 ±10.1 ^ab^	23.87 ± 0.20 ^b^
**BG 280 C**	21.14 ± 4.2 ^ab^	28.41 ± 12.9 ^ab^	26.19 ± 0.05 ^d^
**BG 280 B**	27.63 ± 4.9 ^c^	33.40 ± 11.2 ^abc^	25.30 ± 0.10 ^c^
**BG 160 C**	16.24 ± 2.3 ^a^	42.80 ±17.6 ^c^	23.29 ± 0.00 ^a^
**BG 160 B**	20.91 ± 5.6 ^ab^	36.30 ± 17.1 ^bc^	23.36 ± 0.07 ^ab^

The letters a–e in the upper index indicates that it belongs to homogeneous groups in columns, between which no statistically significant differences were found (*p* < 0.05).

## Data Availability

Data are contained within the article.
